# Tongue function and swallowing in individuals with temporomandibular disorders

**DOI:** 10.1590/1678-7757-2019-0355

**Published:** 2020-04-03

**Authors:** Raquel Rodrigues Rosa, Mariana da Rocha Salles Bueno, Renata Resina Migliorucci, Alcione Ghedini Brasolotto, Katia Flores Genaro, Giédre Berretin-Felix

**Affiliations:** 1 Universidade de São Paulo Faculdade de Odontologia de Bauru Departamento de Fonoaudiologia BauruSão Paulo Brasil Universidade de São Paulo, Faculdade de Odontologia de Bauru, Departamento de Fonoaudiologia, Bauru, São Paulo, Brasil.; 2 Universidade de São Paulo Hospital de Reabilitação de Anomalias Craniofaciais BauruSão Paulo Brasil Universidade de São Paulo, Hospital de Reabilitação de Anomalias Craniofaciais, Bauru, São Paulo, Brasil.

**Keywords:** Tongue, Deglutition, Temporomandibular Joint Disorders

## Abstract

The tongue participates in the oral phase of swallowing by pushing the food bolus toward the oropharynx. This relationship between tongue function and swallowing is little addressed addressed in individuals with temporomandibular dysfunction (TMD).

**Objective:**

To analyze the association of functional tongue conditions on swallowing in individuals with TMD.

**Methodology:**

After approval by the Institutional Review Board, the study was conducted on 30 individuals of both sexes, aged 18 to 28 years, with TMD, and not treated for the disorder. Tongue function was assessed as to the mobility, pressure, and oral motor control. Swallowing was analyzed by clinical assessment during ingestion of solid (wafer biscuit) and liquid (water). Data regarding mobility and swallowing were collected using the orofacial myofunctional evaluation protocol. Tongue pressure was measured by the Iowa Oral Performance Instrument, during elevation, protrusion, swallowing, and resistance test. The oral motor control was assessed by the oral diadochokinesis (DDK) test by rapid and repeated emissions of syllables “ta” and “ka”. Data were statistically analyzed by the Spearman correlation coefficient, at a significance level of 5%.

**Results:**

Relationships were found between tongue function and swallowing for the following aspects: mobility (r=0.741), pressure in protrusion (r=-0.366), swallowing of saliva (r=-0.499), mean DDK rate in emissions “ta” (r=-0.424) and “ka” (r=-0.446), and mean DDK period in emissions “ta” (r=0.424) and “ka” (r=0.446). Thus, the greater the change in tongue mobility, the lower the tongue pressure in protrusion and swallowing of saliva, the lower the emissions per second, the longer the mean time between vocalizations, and the worse the swallowing of individuals with TMD.

**Conclusion:**

The functional conditions of the tongue regarding mobility, pressure, and oral DDK were associated with swallowing in individuals with TMD.

## Introduction

The tongue and the temporomandibular joint (TMJ) are components of the stomatognathic system, and the musculature regarding the action of these structures should work in coordination for the adequate performance of orofacial functions, such as swallowing.^1^ In this function, the tongue participates in the oral phase by pushing the food bolus toward the oropharynx, applying force against the palate with sufficient magnitude and timing, initially at the anterior and then at the posterior region.^2^^–^^5^ This pressure against the palate developed by the tongue is influenced by the food bolus consistency^6^^,^^7^ and is temporally related with the movement of hyoid and mandible during the process.^8^^,^^9^

This description of the tongue function in orofacial functions refers to the normal physiological conditions. However, one of the clinical conditions that impair the stomatognathic system is the temporomandibular dysfunction (TMD), which is related with a combination of alterations affecting the TMJ, masticatory muscles, and associated structures.^10^

The TMD may cause compensations and adaptations to the stomatognathic functions when present,^11^^,^^12^ indicating the need to understand the myofunctional alterations by detailed analysis, to define the adequate treatment planning.^13^ Few studies have analyzed the swallowing in this pathological condition, evidencing atypical swallowing and^14^^,^^15^ alteration in oral^16^ and pharyngeal^17^ phases of swallowing in this population.

As mentioned, the tongue plays a fundamental role in the swallowing function, and little is known about this musculature in individuals with TMD. Recently, physiological changes in the suprahyoid musculature are associated with worse myofunctional condition.^18^ Thus it is important to include other functional measures to better understand this relationship and develop a favorable therapeutic planning for the success of myofunctional therapy.

Therefore, this study analyzed whether functional conditions of the tongue and swallowing are interrelated in individuals with TMD. Considering that individuals with TMD present myofunctional alterations, the hypothesis of this study is that alterations in tongue function regarding mobility, pressure, and oral motor control are related to change in the performance of swallowing in this population.

## Methodology

### Casuistry

The study was conducted after approval by the Institutional Review Board (report no. 703.214/2014, CAAE no. 32231114.1.0000.5417), and all participants signed an informed consent form.

The sample was composed of 30 individuals aged 18 to 28 years, with 24 females and 6 males (median age=23.5 and 24 years, respectively). The individuals were diagnosed with temporomandibular dysfunction according to the Research Diagnostic Criteria for Temporomandibular Disorders (RDC/TMD) protocol, Axis I,^19^ adapted to the Portuguese language.^20^ All individuals were not being treated for the problem and had disc displacement with reduction. Besides this articular disorder, 18 of them also had myalgia (myofascial pain, n=12; myofascial pain with limited mouth opening, n=6).

The inclusion criteria comprised good general health, with at least 28 permanent teeth. The study excluded individuals with periodontal disorders, relevant malocclusion (anterior open bite, posterior or anterior crossbite), history of central or peripheral neurological disorders, surgeries and/or tumors or traumas to the head and neck region, history of speech, physical or orthodontic therapy ongoing or less than one year before this study, presence of pacemakers, chronic intake of analgesic, anti-inflammatory or psychotropic drugs, and pregnancy in the case of females.

Among the individuals, seven had searched for treatment and were waiting for onset of intervention, while the others noticed some symptoms of TMD and volunteered to participate in the study. The severity of TMD signs and symptoms observed by the application of the Protocol for Multi-Professional Centers for the Determination of Signs and Symptoms of Temporomandibular Disorders (ProTMDMulti - Part II)^21^ indicated mild to moderate symptomatology (median total score of 23.5 points – minimum 3 and maximum 112).

### Procedures

Tongue function was assessed as to the mobility, pressure, fine oral motor control, and swallowing by clinical evaluation. The analyses are shown in detail below. During the procedures, the individuals were maintained comfortably seated on a fixed chair, with the feet on the ground, and 90° angle between the hip and knee joints.

Data related to tongue mobility, oral motor control, and swallowing were collected by the MBGR Orofacial Myofunctional Examination Protocol [adapted from Marchesan, Berretin-Felix and Genaro^13^ (2012)] and dynamically recorded using a digital camera (Sony Electronics Inc.; San Diego, California, USA) supported on a tripod at one meter from the individual. The video recordings of mobility and swallowing were separately analyzed by two examiners with clinical and scientific experience in the field, previously calibrated for the procedure. The reliability between examiners, assessed by kappa statistics, was good with almost perfect inter-examiner agreement (k=0.89) and moderate to almost perfect intra-examiner agreement (k=0.46 to 0.97).^22^

### Tongue function

Tongue mobility was assessed during movements of protrusion, touching the apex sequentially at the commissures (right and left), center of lips (upper and lower) and cheeks (right and left), touching the apex on the incisive papilla, clicking the apex, sucking the tongue on the palate, and vibration. Each movement was classified as adequate (0), altered (1), or absent (2) and the sum of scores could range from zero (best result) to 16 (worst result).

Tongue pressure was measured by the Iowa Oral Performance Instrument (IOPI) model 2.2 (IOPI Medical LLC; Carnation, Washington, USA), which contains a light mode display and a bulb, which was positioned in the oral cavity and pressed by the tongue. The tests included: a) elevation, with bulb pressure on the incisive papilla region for 2 seconds; b) protrusion, with the bulb attached to a wooden spatula and located between the incisors, for 2 seconds; c) swallowing, with the bulb located on the incisive papilla and individuals swallowing the saliva as usual; and d) resistance test, pressing the bulb on the incisive papilla region, maintaining 50% of the pressure obtained in the elevation test with monitoring by the equipment light, recording the time in seconds of the pressure maintained. Three consecutive measurements were obtained for all tests, with one-minute rest intervals between them, considering the highest values obtained – recorded in kilopascal (kPa).

The oral motor control was analyzed by oral DDK test by fast and repeated emissions of “ta” and “ka” syllables. The records of emissions were videotaped, and the videos were edited in the Sound Forge Pro 10.0 software (Sony Creative Software Inc.; Middleton, Wisconsin, USA). The first and last three seconds of the sample were removed from each emission, maintaining the emissions performed for 4 seconds. Data from oral DDK were analyzed on the Motor Speech Profile Advanced software, model 5141, version 2.5.2 (KayPENTAX Inc.; Lincoln Park, New Jersey, USA), using the following parameters: mean DDK rate, mean DDK period, standard deviation of DDK period, coefficient of variation of DDK period, jitter in DDK period, and coefficient of variation of DDK peak intensity.

### Swallowing

Swallowing was analyzed during ingestion of solid and liquid. For solid swallowing, a chocolate-flavored wafer biscuit (Bauducco Pandurata Alimentos LTDA; Guarulhos, São Paulo, Brazil) was used, and the individuals were instructed to eat the biscuit as usual. Fluid swallowing comprised 100 mL of water, and the individuals were asked to place a volume of water in the mouth (usual amount), lower the glass, and swallow when requested, three times.

The analysis considered some aspects investigated by two examiners, who assigned scores to each of them: lip posture: (0) closed, (1) partially closed/lower lip contact with upper teeth, or (2) open; tongue posture: (0) not visible/behind the teeth, (1) against the teeth, or (2) between the teeth; food/liquid holding: (0) adequate, (1) partial, or (2) inadequate; contraction of orbicular oris and chin muscles: (0) adequate/absent, (1) little, or (2) marked; head movement, noise, and residue after swallowing: (0) absent or (1) present; coordination: (0) adequate or (1) choking/coughing. The sum of liquid and solid swallowing scores ranged from zero (best result) to 28 (worst result).

### Statistical analysis

Data were analyzed by descriptive statistics and the Spearman correlation test at a significance level of p<0.05, using the SigmaPlot 12.0 software (Systat Software Inc.; Chicago, Illinois, USA) to analyze the relationship between tongue function and swallowing. The correlations were classified according to the r value: weak (0.10 to 0.30), moderate (0.40 to 0.60), or strong (0.70 to 1).^23^

## Results

The scores obtained in the assessment of tongue function regarding mobility (median=0, minimum=0, and maximum=5), as well as those observed in swallowing (median=1.5, minimum=0, and maximum=5) were low, indicating little change according to the protocol used. The tongue pressure values and oral DDK are shown in Table 1.

**Table 1:** Values of tongue pressure in kilopascals (kPa), in elevation, protrusion, swallowing and resistance tests, and oral DDK values in emissions “ta” and “ka”

Tests		Mean	Standard deviation
Tongue pressure (kPa)	Elevation	56.333	11.260
	Protrusion	44.133	11.927
	Swallowing	34.333	14.667
	Resistance test	19.267	9.854
Oral diadochokinesis			
avr (emission/s)	“ta”	5.905	0.862
	“ka”	5.516	0.831
avp (ms)	“ta”	172.800	24.680
	“ka”	185.266	27.687
sdp (ms)	“ta”	23.221	16.146
	“ka”	30.636	18.497
cvp (%)	“ta”	13.468	8.968
	“ka”	16.589	9.836
jit (%)	“ta”	2.709	1.784
	“ka”	3.148	1.717
cvi (%)	“ta”	3.045	0.946
	“ka”	3.337	0.935

avr=average DDK rate; avp=mean DDK period; sdp=standard deviation of DDK period; cvp=coefficient of variation of DDK period; jit=jitter of DDK period; cvi=coefficient of variation of DDK peak intensity; s=seconds; ms=milliseconds; %=percentage

Despite the few alterations observed, a relationship was found between tongue function and swallowing (Table 2). It showed a strong positive correlation with mobility, indicating that, with the alteration in mobility, the worse the swallowing performance. There was also weak negative correlation between tongue pressure in protrusion and the score in swallowing, as well as moderate negative correlation between tongue pressure in swallowing of saliva and the score in swallowing, demonstrating that the lower the tongue pressure, the worse the performance of swallowing for individuals with TMD.

**Table 2:** Correlation coefficient values between functional conditions of the tongue (mobility and pressure), oral DDK in emissions “ta” and “ka”, and swallowing function

Score in swallowing X			r value	P value
Tongue Mobility			0.741*	<0.001*
Tongue Pressure				
	Elevation		-0.096	0.610
	Protrusion		-0.366*	0.047*
	Swallowing		-0.499*	0.005*
	Resistance test		0.245	0.189
Oral diadochokinesis				
avr (emission/s)		“ta”	-0.424*	0.020*
		“ka”	-0.446*	0.014*
avp (ms)		“ta”	0.424*	0.020*
		“ka”	0.446*	0.014*
sdp (ms)		“ta”	0.234	0.211
		“ka”	0.251	0.179
cvp (%)		“ta”	0.081	0.667
		“ka”	0.191	0.309
jit (%)		“ta”	0.254	0.173
		“ka”	0.298	0.109
cvi (%)		“ta”	0.300	0.106
		“ka”	0.194	0.302

* relationship between tongue function and swallowing, according to the Spearman correlation test (p<0.05).

avr=average DDK rate; avp=mean DDK period; sdp=standard deviation of DDK period; cvp=coefficient of variation of DDK period; jit=jitter of DDK period; cvi=coefficient of variation of DDK peak intensity; s=seconds; ms=milliseconds; %=percentage

Table 2 shows the relationship between oral DDK and score in swallowing, indicating moderate negative correlation with the mean DDK rate and positive with the mean DDK period for both emissions. Thus, the lower the emissions *per* second and the highest the mean duration of vocalizations, the worse the swallowing function.

## Discussion

This study investigated the association between tongue function and swallowing in individuals with TMD, considering the importance to understand this relationship to allow adequate therapeutic planning. This process included clinical and instrumental analyses, using scientifically proven instruments that provide reliable values.^24^

Individuals included in the sample presented specific characteristics regarding the diagnosis and degree of severity of signs and symptoms of the dysfunction, which might have impacted the results observed. Among the 30 individuals, some searched for treatment and were waiting for the onset of intervention, with long-term complaint (minimum 18 and maximum 110 months); the others were volunteers from the community that noticed some symptoms, especially clicking, and most could not report the duration.

All participants were classified with disc displacement with reduction. This type of disorder has been considered the most common among intraarticular alterations, and its clinical repercussion is discussed because there may be adaptation of the TMJ structures without the need for treatment, unless the patient presents some complaint.^25^ However, no long-term studies assessed and followed the performance of these individuals' tongue and swallowing function to confirm the orofacial myofunctional adjustments in this population. Also, the degree of TMD severity was low, indicating mild symptomatology.

The aforementioned characteristics may justify the low score observed in the assessment of tongue mobility, as well as swallowing, since in studies that found myofunctional alteration, the included individuals were patients searching for treatment^11^^,^^12^^,^^17^ and presenting dysfunction for a long time.^18^^,^^26^^,^^27^

Regarding the tongue pressure, the values found in the protrusion and saliva swallowing analysis in this study were greater than the ones observed in individuals with TMD.^27^ This difference may be related to the characteristics of involved individuals, since in the mentioned study the patients presented worse symptomatology and chronic TMD. Also, the values obtained were similar to those observed in Brazilian individuals without TMD concerning the elevation, swallowing, and especially for females in protrusion and the resistance test, since the values were reduced in both tests compared with males.^28^

However, most participants in this group were females. According to the meta-analysis performed by Adams, et al.^24^ (2013), tongue force in males is higher than in females, with discrepancy of 5.21 kPa. The similarity of tongue pressure values found and those reported in the literature suggests that, for individuals in this study, the TMD did not impact the tongue pressure, probably because of the participants' aforementioned characteristics.

For comparison of the DDK results, no data regarding the TMD were found in the literature. Therefore, the observations were performed with studies published in Brazilian adults. The mean rate and mean period were similar to the non-dysphonic females in the study of Louzada, et al.^29^ (2011), and lower than observed by Padovani, Gielow, and Behlau^30^ (2009). However, the coefficient of variation of the period, jitter of the period, and coefficient of variation of the peak intensity were higher than in the mentioned literature. Concerning the oral motor control, these observations suggest individuals with TMD showed worse ability to keep constant emissions for seconds, i.e. instability in the oral motor control involving the tongue. Therefore, this aspect should be investigated and considered in the process of orofacial myofunctional therapy when indicated.

In the analysis of relationships between tongue function and swallowing, the aim of this study, the positive correlation found between tongue mobility and swallowing indicates that the alteration in mobility leads to worse performance in swallowing. In the transportation of the food bolus to the oropharynx, during the force applied against the palate, the tongue performs anteroposterior movement for effective propulsion^2^^–^^4^ and the instability of this structure to perform isolated movements may have functional consequences.^11^

The tongue pressure in protrusion and swallowing of saliva was negatively correlated with the swallowing function for individuals with TMD. Therefore, individuals with TMD with reduced tongue pressure had greater difficulty in performing the swallowing function adequately. This relationship was also observed in the study of Marim, et al. (2019), who observed that reduced tongue pressure can contribute to alter the swallowing pattern, thus these aspects should be considered in the clinical practice.^27^

The lower speed of repetition of syllables “ta” and “ka”, consequently with higher mean of the period of these repetitions, may be associated with worse control of tongue movements.^30^ The negative correlation between the score in the assessment of swallowing and the mean DDK rate, and the positive correlation with the mean DDK period indicated that, with the alteration in swallowing, fewer emissions were produced *per* second and the longer the mean time was between vocalizations, for both the anterior and posterior tongue regions, by the emission of “ta” and “ka”, respectively. Since the tongue contact with the palate is synchronically performed on the anterior, middle, and posterior regions during swallowing,^2^^–^^4^ the alteration in the oral motor control regarding the tongue may impair the performance of swallowing in individuals with TMD.

As observed, there was association between tongue function regarding mobility, pressure and oral DDK with swallowing. Considering that the literature indicates the occurrence of myofunctional disorder as a compensatory mechanism of the altered stomatognathic system in the presence of TMD,^11^^,^^12^ even though individuals in this study did not present significant damage to the analyzed functions, maybe, because of the associations observed, they might be performing myofunctional adaptations of the stomatognathic system to achieve better functional performance, despite the mild symptoms.

Further studies should be conducted to continue this investigation, considering groups with specific dysfunction classifications, separately analyzed, as well as compared with a control group, and should follow these individuals in the long-term to investigate the functional adjustments performed and confirm the impact on orofacial functions.

Even though no important damage was observed on the tongue and swallowing functions compared with the literature, when these aspects were related, an association was observed for tongue mobility and pressure and oral DDK. Thus, when therapeutic intervention is indicated for a patient with characteristics that are similar to those of this study, these aspects may be considered when assessing the treatment plan, especially for orofacial myofunctional therapy.

## Conclusion

The functional conditions of the tongue regarding mobility, pressure, and oral DDK was associated with swallowing in individuals with TMD.
